# Rocuronium-induced neuromuscular block and sugammadex in pediatric patient with duchenne muscular dystrophy: A case Report

**DOI:** 10.1097/MD.0000000000006456

**Published:** 2017-03-31

**Authors:** Ji Eun Kim, Hea Rim Chun

**Affiliations:** Department of Anesthesiology and Pain Medicine, Soonchunhyang University Hospital Cheonan, Dongnam-gu, Cheonan, Chungcheongnam-do, Korea.

**Keywords:** duchenne muscular dystrophy, neuromuscular blocking agents, pediatrics, sugammadex

## Abstract

**Introduction:**

Anesthetic management of patients with Duchenne muscular dystrophy (DMD) is complicated because these patients are more sensitive to nondepolarizing neuromuscular blocking agents (NMBAs) and are vulnerable to postoperative complications, such as postoperative residual curarization and respiratory failure. Sugammadex is a new reversal agent for aminosteroidal NMBAs, but its safety in children is controversial.

**Clinical features:**

An 11-year-old boy with DMD underwent general anesthesia for a percutaneous nephrolithotomy. We used rocuronium bromide and sugammadex to reverse the deep neuromuscular block. Reversal of neuromuscular block was done 15 minutes after administration of 2 mg/kg of sugammadex. The patient's recovery from anesthesia was uneventful, and he was discharged to the postoperative recovery ward.

**Conclusion:**

A delayed recovery was achieved, but no adverse events were observed, such as recurarization or hypersensitivity to sugammadex. We report safe use of 2 mg/kg of sugammadex to reverse a deep neuromuscular block in a child with DMD.

## 1 Introduction

Duchenne muscular dystrophy (DMD), an X-linked recessive disease and the most common and severe type of muscular dystrophy, has an incidence of 1 per 3500 to 5000 male births.^[1,2]^ The defect is located on the short arm of the X chromosome at the Xp21 region; this region contains the dystrophin gene,^[2,3]^ which is expressed in skeletal, smooth, and cardiac muscle, as well as in the brain.^[4]^ Dystrophin plays an important role in stabilizing the sarcolemma and maintaining muscle membrane integrity. Lack or dysfunction of dystrophin leads to fragility of the sarcolemma and increased membrane permeability.^[5]^ The common signs and symptoms at presentation include a waddling gait, calf hypertrophy, and the classic Gowers sign because of proximal muscle weakness. Serum creatine kinase (CK) and hepatic transaminase levels are elevated.^[1]^

Cardiomyopathy and arrhythmias occur in patients with DMD because of degeneration of cardiomyocytes.^[1]^ Because pulmonary insufficiency is a common cause of morbidity and mortality in patients with DMD,^[6]^ preoperative pulmonary assessment is required. Progressive decline in pulmonary function is a hallmark of the disease; thus, the majority of deaths in patients with DMD are because of pulmonary causes.

Succinylcholine, which is a depolarizing neuromuscular blocking agent (NMBA), is contraindicated in patients with DMD because of the potential for rhabdomyolysis, hyperkalemia, and hyperkalemic cardiac arrest as a result of unstable sarcolemmal membranes.^[3]^ The use of volatile anesthetics should be avoided in these patients,^[3,7]^ and most experts advise using total intravenous anesthesia.^[8]^ Patients with DMD tend to have increased sensitivity to the effects of a nondepolarizing NMBA at a given dose, so that an increase in both the maximal effect and duration of action usually accompanies administration of a nondepolarizing NMBA.^[9,10]^

Sugammadex reverses rocuronium- and vecuronium-induced neuromuscular block (NMB). Case reports of patients with myasthenia gravis have documented 117 cases of successful use of sugammadex,^[11]^ but reports on rare muscular diseases, such as DMD, have documented only 2 cases of successful reversal of rocuronium, with 4 mg/kg sugammadex in a child ^[12]^ and 2 mg/kg sugammadex in an adult.^[13]^

In this case, we report the use of 2 mg/kg of sugammadex to reverse a deep NMB in a child with DMD.

## 2 Case report

An 11-year-old boy, weight 53 kg, with a ureter stone was scheduled for percutaneous nephrolithotomy under general anesthesia. He was diagnosed with DMD at the age of 1 year and was in a bed-ridden state recently. Preoperative evaluation revealed an abnormal electrocardiogram (ECG) finding (Right ventricular hypertrophy and rSR on V1) and elevated CK, aspartate aminotransferase, and alanine aminotransferase (ALT) levels.

Glycopyrrolate 0.2 mg was injected intramuscularly as premedication. On arrival at the operating room, standard intraoperative monitoring, including ECG, pulse oximetry, and noninvasive arterial blood pressure, was performed.

Train-of-four (TOF) stimuli were applied to the ulnar nerve by monitoring recovery of NMB using an electromyographic neuromuscular transmission module (M-NMT Module; Datex-Ohmeda Inc, Helsinki, Finland). Recovery of the TOF ratio (%) to 90% was considered as reversal of the NMB.

After preoxygenation, anesthesia was induced with 5 mg/kg of pentothal sodium and 5 mg of midazolam, and maintained with continuous intravenous (IV) infusion of 250 μg/kg/min of propofol and 0.3 μg/kg/min of remifentanil. The initial TOF ratio (%) was 86% and the TOF count was 4 before the patient received an IV bolus injection of 0.6 mg/kg rocuronium bromide. After endotracheal intubation, the lungs were ventilated with a 1:2 mixture of oxygen and air, and the left radial artery was cannulated after the modified Allen's test was done to monitor invasive blood pressure. One hour after induction, the operation was started; 10 mg rocuronium bromide was injected IV 110 minutes after induction because the TOF count and ratio were 4 and 15%, respectively. The durations of the operation and anesthesia were 90 minutes and 3 hours, respectively. At the end of the procedure, neuromuscular monitoring showed a TOF ratio of 0% and a TOF count of 0, indicating deep NMB. Reversal of the rocuronium-induced NMB was performed by administering 2.0 mg/kg sugammadex (106 mg). We obtained a TOF ratio of 71% within 260 seconds, which increased to 90% after 10 minutes. No clinically relevant changes from baseline were observed in arterial blood pressure or heart rate after administration of sugammadex. Tracheal extubation was done 15 minutes after administration of sugammadex. No signs of adverse effects, such as parosmia or hypersensitivity, were observed after sugammadex was administered. The patient's recovery from anesthesia was uneventful, and he was discharged to the postoperative recovery ward. No signs of residual NMB or recurarization were observed, and the patient was discharged to the ward 1 hour later.

## 3 Discussion

Several studies have documented a markedly prolonged NMB following administration of different nondepolarizing NMBAs in patients with DMD.^[9,10]^ Administration of cholinesterase, the usual method to reverse rocuronium, has limitations because of the wide variability in recovery times among patients with DMD.^[14]^

Sugammadex (Bridion; Merck & Co, Whitehouse Station, NJ) is a gamma-cyclodextrin ^[15]^ used to reverse nondepolarizing NMBAs. It encapsulates and inactivates aminosteroidal NMBAs, such as rocuronium and vecuronium. Although sugammadex is a safe drug,^[16]^ it has several side effects, such as coughing, involuntary movements of the limbs or body, parosmia,^[17]^ and hypersensitivity, which are rare but important reactions.^[18]^ Although sugammadex has not been approved by the US Food and Drug Administration for use in children, many studies have been published about the safe and efficient use of 2 to 4 mg/kg of sugammadex in pediatric patients.^[19,20]^ The recommended dose for adults seems to be equally efficient but has a faster onset time, with a very narrow range of individual responses in children.^[19]^ Therefore, the recommended dose of sugammadex in pediatric patients is 2 mg/kg for a moderate block. Nevertheless, successful reversal of a rocuronium-induced deep block with 2 mg/kg sugammadex has been reported in a young child.^[21]^ In our case, the TOF counts and ratios, at the end of the surgery and 40 minutes after the second administration of 10 mg rocuronium bromide, were both 0, indicating a deep block. Therefore, we initially planned to use 2 mg/kg of sugammadex, and to use an additional 2 mg/kg if signs of postoperative residual curarization appeared. Although we obtained a TOF ratio of 71% 260 seconds after administration of sugammadex, and the TOF ratio reached 90% 10 minutes later, PORC and a reduced TOF ratio did not appear after 15 minutes, and he had no signs of adverse effects after administration of sugammadex.

A few studies are available about the safe use of sugammadex in patients with DMD.^[12,13]^ In our case, reversal of deep NMB by sugammadex was delayed but safe, and no PORC occurred. Although more cases should be documented, we recommend 2 mg/kg of sugammadex to reverse deep NMB in children with DMD.
